# Revising Alpine summer temperatures since 881 CE

**DOI:** 10.1007/s00382-024-07195-1

**Published:** 2024-03-22

**Authors:** Eileen Kuhl, Jan Esper, Lea Schneider, Valerie Trouet, Marcel Kunz, Lara Klippel, Ulf Büntgen, Claudia Hartl

**Affiliations:** 1 Department of Geography, Johannes Gutenberg University, Johann-Joachim-Becher Weg 32, 55128 Mainz, Germany; 2 Department of Geography, Justus-Liebig-University, Gießen, Germany; 3 Laboratory of Tree-Ring Research, University of Arizona, Tucson, USA; 4 Deutscher Wetterdienst, Offenbach, Germany; 5 Department of Geography, University of Cambridge, Cambridge, UK; 6 Nature Rings - Environmental Research and Education, Mainz, Germany; 7 Global Change Research Centre (CzechGlobe), Brno, Czech Republic; 8 Swiss Federal Research Institute (WSL), Birmensdorf, Switzerland; 9 Department of Geography, Masaryk University, Brno, Czech Republic

**Keywords:** Climate reconstruction, Climate change, Dendrochronology, Europe, European Alps, Tree rings

## Abstract

Europe experienced severe heat waves during the last decade, which impacted ecological and societal systems and are likely to increase under projected global warming. A better understanding of pre-industrial warm-season changes is needed to contextualize these recent trends and extremes. Here, we introduce a network of 352 living and relict larch trees (*Larix decidua* Mill.*)* from the Matter and Simplon valleys in the Swiss Alps to develop a maximum latewood density (MXD) chronology calibrating at r = 0.8 (p > 0.05, 1901–2017 CE) against May–August temperatures over Western Europe. Machine learning is applied to identify historical wood samples aligning with growth characteristics of sites from elevations above 1900 m asl to extend the modern part of the chronology back to 881 CE. The new Alpine record reveals warmer conditions in the tenth century, followed by an extended cold period during the late Medieval times, a less-pronounced Little Ice Age culminating in the 1810s, and prolonged anthropogenic warming until present. The Samalas eruption likely triggered the coldest reconstructed summer in Western Europe in 1258 CE (-2.32 °C), which is in line with a recently published MXD-based reconstruction from the Spanish Pyrenees. Whereas the new Alpine reconstruction is potentially constrained in the lowest frequency, centennial timescale domain, it overcomes variance biases in existing state-of-the-art reconstructions and sets a new standard in site-control of historical samples and calibration/ verification statistics.

**Supplementary Information:**

The online version contains supplementary material available at 10.1007/s00382-024-07195-1.

## Introduction

There is a rising urgency to improve our understanding of natural and anthropogenic drivers of temperature variations and reduce uncertainties in models predictions (Eyring et al. 2019). Analysing past climate variability, identifying spatial patterns and updating paleoclimate models is indispensable to establish the basic conditions for these models (Esper and Büntgen 2021). Common paleoclimate archives are tree-rings from high-elevation and high-latitude sites, which are used to reconstruct past temperature variability and place anthropogenic warming into historical context (Schweingruber 1988). This is not only true for long-term changes in climate history but for annual fluctuations, for example, exceptionally warm/cold and wet/dry years. With information on extreme events, we can set recent extreme events (and the likelihood of future events) in a historical context regarding their timing, occurrence and strength (e.g., Qin et al. 2011; Lyu et al. 2016; Borkotoky et al. 2021).

The European Alps have been of interest for such studies for decades as the regional warming already exceeded 2 °C from 1864 to 2017 CE (Allgaier Leuch et al. 2017). Tree-ring width (TRW) and maximum latewood density (MXD) have been used as proxies to develop eleven Alpine temperature reconstructions (Schweingruber et al. 1987, 1988; Büntgen et al. 2005, 2006; Frank et al. 2005; Frank and Esper 2005a; Esper et al. 2007b; Corona et al. 2010, 2011; Trachsel et al. 2012; Coppola et al. 2013; Leonelli et al. 2016; Table 1). Mainly, these publications used larch (*Larix decidua* Mill.*)* tree-ring series with one exception, which is a spruce (*Picea abies* L.*)* reconstruction (Esper et al. 2007b).

**Table 1:** Existing TRW- or MXD-based temperature reconstructions based on from the European Alps in order of publication year. The table includes the number of included sites, the elevation, the used proxies, the range of years covered, as well as the species (Larix decidua Mill. = LADE, Abies alba Mill. = ABAL, Pinus cembra L. = PICE, Picea abies L. = PIAB) and the temperature signal used for reconstructing (letters denote to the first letter of the corresponding months, e.g. JJA June, July, August)

No	Publication	# of Sites	Elevation [m asl]	Proxy	Period [CE]	Species	Temperature Signal
1	Schweingruber et al. 1987, 1988^h^	16	100–1700, 1800	MXD	982–1976	PIAB, ABAL	JJAS
2	Büntgen et al. 2005^h^	5	> 1500	TRW	951–2002	LADE, PICE	JJA
3	Frank and Esper 2005a, b	53	> 1500	TRW / MXD	1600–1988/ 1650–1987	PIAB, ABAL, LADE, PICE	JJA / AMJJAS
4	Frank et al. 2005	53	> 1500	TRW	1760–1990	PIAB, ABAL, LADE, PICE	JJA
5	Büntgen et al. 2006^h^	4	> 1900	MXD	755–2004	LADE	JJAS
6	Esper et al. 2007b^h^	4	-	TRW /MXD	1028–2003	PIAB	JJ / AS
7	Corona et al. 2010^h^	38	> 1725	TRW & MXD	1000–2000	LADE, PICE	JJA
8	Corona et al. 2011^h^	34	> 1600	TRW	751–2003	LADE	JJA
9	Coppola et al. 2013	4	> 1910	TRW	1610–2008	LADE	JJA
10	Trachsel et al. 2012^h^	4	-	TRW, MXD, BI, Chrionomid/ biogenic Silica	755–2004	LADE, PIAB, PICE	JJA
11	Leonelli et al. 2016	42	> 1800	TRW	1470–2010	LADE, PIAB, PICE	JJA

^h^ = used historical series from buildings

Most analyses targeted summer temperatures from June to August (JJA), whereas a few MXD chronologies were used for longer seasons: Schweingruber et al. (1987, 1988) and Büntgen et al. (2006) calibrated their MXD chronologies to June to September (JJAS) temperatures, while Frank and Esper (2005a, b) reconstructed April to September temperatures. One exception is Esper et al. (2007b), which found best correlations with temperatures from June-July for TRW and August–September for MXD. Seven of the eleven reconstructions extend back to the first millennium CE (Schweingruber et al. 1987, 1988; Büntgen et al. 2005, 2006; Esper et al. 2007b; Corona et al. 2010, 2011; Trachsel et al. 2012). MXD-based chronologies reached correlations up to 0.69 with instrumental JJAS temperatures (Büntgen et al. 2006). The most recent reconstruction reported correlations up to 0.78 with JJA from 1763 to 2014 CE (Leonelli et al. 2016). Out of all mentioned publications, Büntgen et al. (2006) received by far the most citations for their reconstruction from the Swiss Lötschental (hereafter referred to as Lötschental) implying the data often being used in other studies and the findings having a generally high impact on the scientific community. Above all, it is the longest existing MXD-based reconstruction from the Alps based on a single species and serves as comparison for this study.

The mentioned millennium-long chronologies all include wood samples from historical buildings (e.g. Corona et al. 2010; Trachsel et al. 2012) as high-elevation forests in the Swiss Alps have been heavily influenced by anthropogenic forest use since the Middle Ages (Conedera et al. 2017). Therefore, the likelihood of living trees spanning earlier ages is low and in situ dead wood in the Alps is rare. The long history of human activity, however, enables the use of construction timber of old, high-elevation settlements like Zermatt in Switzerland (Coolidge 1912). The recurring issue with historical tree-ring samples in all of the existing reconstructions spanning a millennium is the limited site control and validation that these woods originate from elevations near treeline and contain strong temperature signals (Riechelmann et al. 2020). Multiple studies showed that the temperature signal in tree-ring parameters weakens with decreasing elevation (Neuwirth et al. 2004; Affolter et al. 2010; King et al. 2013; Hartl-Meier et al. 2014a, b, 2017b; Salzer et al. 2014; Zhang et al. 2015; Hartl et al. 2022), which can consequently influence the temporal robustness of a reconstruction. Kuhl et al. (2023) recently introduced a method to mitigate these biasing effects by fitting tree-ring parameters and growth characteristics to a classification model to sort historical series to living stands with different distances to treeline. This provenance method was tested on data from the Swiss Simplon Valley in Kuhl et al. (2023) and is now applied to the Swiss Matter Valley data as well to exclude historical series with growth behaviours similar to sites further away from the current treeline. With this approach, we aim to build an improved millennium-length temperature reconstruction by the selection of historical material based on the information we gain from the provenance models.

We present a new MXD-based temperature reconstruction from 352 living and historic larch trees* (Larix decidua* Mill*.)* sampled in the southwestern Swiss Alps. We analyse the influences of detrending and variance stabilisation on reconstruction variability and trends, assess and mitigate the effects of larch budmoth infestation events, and revise Alpine temperature history since 881 CE including severe changes compared to existing reconstructions from the European Alps. The record is compared with other high-resolution warm season temperature reconstructions, and common trends and extremes are discussed to evaluate large-scale climate forcings.

## Methods

### Study sites and data distribution

**Fig. 1:**
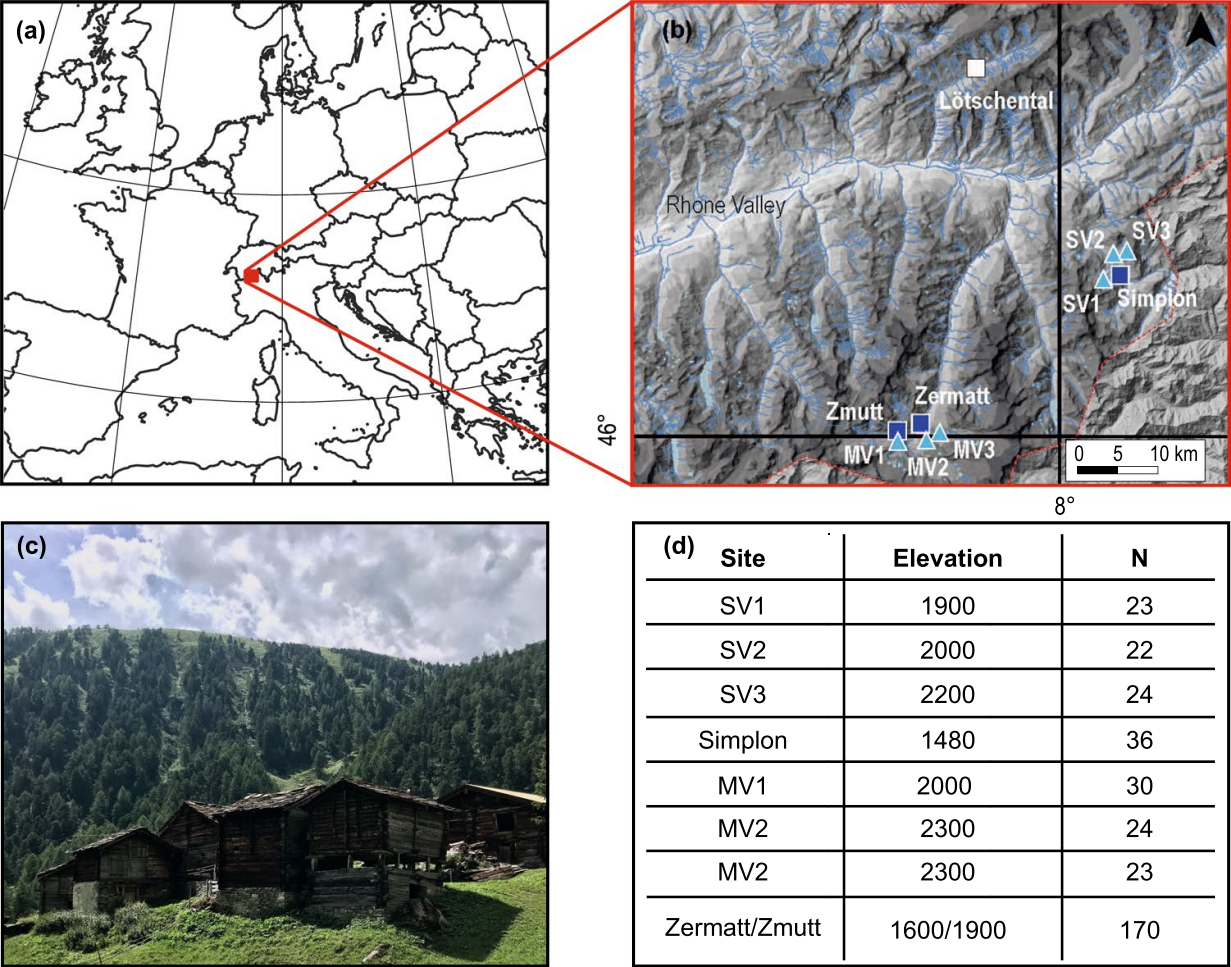
**a** Study site in the Swiss Alps close to the Italian border, **b** and locations of the sampling sites for living trees (turquoise triangles) and historical buildings (dark blue quarters) in the Matter Valley (MV) and the Simplon Valley (SV) **c** The historical village of Zmutt in the Matter Valley (credits: C. Hartl) **d** Information about the elevation [m asl] and the number of samples (increment cores and discs) per site

**Fig. 2:**
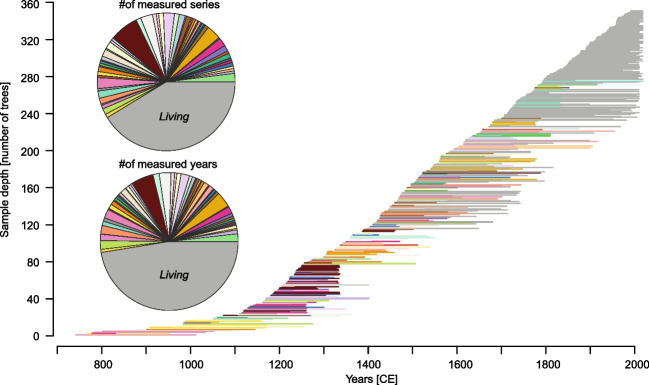
Temporally balanced distribution of the 146 living (grey) and 206 relict (coloured) samples, sorted by their innermost rings in a bar plot. Pie charts show the even distribution of samples per house (top) and the total number of yearly measurement points per building (bottom). Both, pie charts and bar plot support a balanced ratio between the number of historical and living measurements

The study sites in the canton Valais in the South-Western Swiss Alps include a total number of 352 series from living trees and historical buildings from valleys south of the Rhone valley (Fig. 1a-c). In the Simplon (SV) and Matter (MV) valleys (Hartl et al. 2022; Kunz et al. 2023), 146 living series of larch (*Larix* *decidua* Mill.) from high elevations (hereafter SV1-3 and MV1-3) were sampled. Likewise, 99 historical series from buildings in Simplon village and 206 historical series from buildings in Zermatt and Zmutt villages (Schmidhalter, M., Riechelmann et al. 2013, 2020) were sampled (Fig. 1b-d). From these samples, X-ray densitometric measurements were conducted using a Walesch2003 (WALESCH, Electronic GmbH, Switzerland) as described in Björklund et al. (2019). The distribution of historical series in time was evaluated to prevent biases arising from an unbalanced dataset (Fig. 2, Esper et al. 2016).

### Provenancing of the historical material

To determine the provenance of the historical series, two Machine Learning (ML) models were trained based on densitometric measurements and statistical data from transect sites in both valleys. For SV, the procedure is described in Kuhl et al. (2023) and was likewise applied to MV. Compared to SV, MV had only a limited number of available sites from different elevations (MV1 and MV2/MV3). Hence, samples showing a prediction probability lower than 0.8 in the ML model output were excluded from the analysis. In rare cases, when A and B samples were predicted to origin from different elevation classes, these were manually assigned to the most likely elevation. From all measured historical samples, 36 (out of 99) from SV and likewise 170 (out of 206) from MV were used for this study. The final datasets were produced by including the historical series to the corresponding living series collectives (Fig. 1c, Table S1).

### Detrending

After provenancing, the sites were mean-adjusted valley by valley to the MXD level of the highest sites (Fig. S1). Differences in mean levels were estimated by calculating the offset between regional curves (RCs) over a period of replication ≥ 15 series (100 cambial years SV, 250 cambial years MV) for each site. The data from the two valleys were then combined and likewise adjusted in their mean levels over the first 300 years of cambial age meeting > 15 series. Missing rings were in-filled using Arstan (Version 41d, Cook 1985), series were power transformed, pruned (Briffa et al. 2001) beyond 300 years of cambial age (considering pith offsets) and standardized in R 4.2 (R Core Team 2021). To remove age-related density changes (Bräker 1981) the series were detrended as residuals (Cook and Peters 1997) using regional curve standardization (RCS, Esper et al. 2003) with a 67% mean cambial age smoothing filter (frequency response = 0.5). In addition, the signal free (SF) approach (Melvin and Briffa 2008) on RCS and age-dependent spline detrending (Melvin et al. 2007),as well as a 300-year smoothing spline (Cook and Peters 1981) and Hugershoff detrending (Cook et al. 1990) were applied for comparison (see Fig. S2). SF detrending was calculated using the program Signal Free (Version 45_v2b, Cook et al. 2017).

### Larch budmoth treatment

Larch trees in these elevations and regions of the European Alps are affected by recurring larch budmoth (*Zeiraphera griseana* Hb., LBM) mass outbreaks, leading to defoliation, and consequently to reduced MXD values and radial growth in the event year (Esper et al. 2007a; Baltensweiler et al. 2008; Hartl-Meier et al. 2016, 2017b; Kunz et al. 2023). These distinct declines in MXD are independent of weather conditions and need to be removed when reconstructing temperature. Several methods were introduced by Büntgen et al. (2009) or Kunz et al. (2023) for LBM outbreak detection. From these methods, impulse indicator saturation (Pretis et al. 2018, IIS) is the most effective one for our purpose as it not only detects LBM events but also gives a correction factor for these years. The raw MXD series were detrended prior to IIS using a 30-year smoothing spline. This algorithm identifies rapid breaks (Fig. S3a) as negative outliers and uses these as correction coefficients to be subtracted from the RCS detrended series (Fig. S3b). The method was initially introduced to accurately detect volcanic events in simulated temperature time series and has proven its potential for LBM detection (Pretis et al. 2017; Schneider et al. 2017; Kunz et al. 2023). When applying IIS, a non-host chronology can be included as a regressor to ensure that the algorithm does not detect outliers that show climate induced declines (e.g. volcanic events) (Kunz et al. 2023). Between 1616 and 2017 CE a non-host Swiss stone pine (*Pinus cembra* L.) site from MV (Kunz et al. 2023) and a non-host larch (*Larix decidua* Mill.) site from the Northern Alps (Hartl-Meier et al. 2014a) were included in the algorithm (Fig. S3c). As the non-host chronologies only extend back to 1616 CE, prior comparison to the host chronology reaching back to 881 CE was prevented. Consequently, the algorithm detected known volcanic events as LBM events in this period. To avoid the correction of climatic induced growth declines, stratospheric aerosol optical depth (SAOD) years from Sigl et al. (2021) were averaged over the Westerlies zone (~ 30–60°N). In years, where SAOD values exceeded 0.03 and matched an IIS detected year, the LBM correction value was not subtracted from the host chronology. The valleys were treated individually to address the lag of outbreak years between the valleys (Johnson et al. 2004; Saulnier et al. 2017; Kunz et al. 2023). Comparison of LBM detections of the IIS algorithm corrected chronology and the non-host chronology (Fig. S3c) confirms that the algorithm can detect non-climate-related growth declines. The prevention of false detection of volcanic cooling events as LBM outliers by using the SAOD values is necessary to preserve climatic signals in the chronology, but it can lead to overestimated cooling when LBM events and volcanic induced cooling years align.

### Variance stabilization

The final chronology was constructed using Tukey’s bi-weight robust mean. To account for changes in variance, chronology variance was stabilized using the method detailed in Osborn et al. (1997). On trial, different window lengths were tested during stabilization. Results showed shorter windows (e.g. 31 years) stabilize variance better than larger ones. However, applying a narrow window for variance stabilization increases the risk to eradicate low-frequency trends (Frank et al. 2007). We approached this risk by calculating residuals between the chronology and a 100-year low-pass Butterworth filter. The variance of the high-frequency residuals was then stabilized in a 31-year window, whereas the low-pass chronology was stabilized in a 201-year window (Osborn et al. 1997)). Afterwards, the stabilized sub-chronologies were added back together to the final chronology from 881 to 2017 CE. Calculations were performed in R 4.2 (R Core Team 2021) using the package “dplR” (Bunn 2010).

### Climate signals

Pearson’s correlations between instrumental temperatures (TS 4.06; Harris et al. 2020) and the MXD chronology were calculated from 1901–2017 CE in a classical bootstrap approach (boot_n_ = 1000, α = 0.95) using the R package “treeclim” (Zang and Biondi 2015). Correlations were computed for monthly January–September (J-S) and JJA and May–September (MJJAS) seasonal means. For every grid from -10°E to 20°W and 35–65°N, we likewise calculated correlations between MJJAS mean temperature anomalies and the tree-ring chronology. We averaged the temperature data of all grids exceeding r = 0.7. Static and running correlations (31-year window) were performed using this averaged temperature record.

### Calibration, verification, and reconstruction

Three different reconstruction approaches (linear regression, polynomial regression, and scaling) were tested using a two-fold cross-validation from 1901–1959 and 1960–2017 CE against MJJAS grid-averaged temperature anomalies. The correlation between predicted and observed data (r), explained variance (R^2^) and root mean squared error (RMSE) were used to assess model accuracies. For the final reconstruction, the best performing model was chosen and then calibrated over the full period from 1901–2017 CE.

Uncertainty bands for the reconstruction were estimated by averaging RMSEs of sub-chronology reconstructions for different, stepwise increasing sample depths from n = 5–50 series in five steps. For each of these replication steps, bootstrapped sub-chronologies of n randomly sampled series were built (boot_n_ = 1000). These individual sub-chronologies were then used to reconstruct MJJAS temperature anomalies on the calibration period 1914–2002 CE. For each reconstruction r, R^2^ and RMSE were calculated. The mean RMSE for each replication step (RMSE_n_) was then used to estimate the error of the reconstruction depending on its sample depth n (error = 2*RMSE_n_).

### Standard reconstruction approach

A reconstruction including all existing historical series was additionally modelled neglecting provenance information. The data were gap-filled, power transformed and pruned (300-years). In contrast to the new approach, no mean adjustment was performed. All series were detrended, LBM corrected, and variance stabilized, just as the main chronology of this paper. A linear model with the same temperature data was used to reconstruct the MJJAS mean temperature variability (averaged grids), and the results compared with the novel provenance considering reconstruction.

### Superposed epoch analysis and extreme year analysis

Superposed epoch analysis (Lough and Fritts 1987, SEA) was applied to evaluate the significance of major volcanic eruptions (Table S2) considering a period of five years prior (reference period) and five years after the event using the package “dplR” (Bunn 2010). For this, a 50-year high-pass filter was run on the new reconstruction and the Lötschental record (Büntgen et al. 2006). From bootstrap resampling (boot_n_ = 10,000), 99% confidence intervals were calculated to estimate significant deviations after volcanic event year period. In addition, the coldest 20 years of the 50-year high-pass filtered records were compared against volcanic event years.

## Results

### Chronology development

The developed chronology covers the period from 881–2017 CE including 1137 years and thus has the potential to revise climate variability back to the early medieval period. The minimum sample depth n = 5 spans the period 881 to 901 CE and the maximum is reached in 1963 CE with n = 93 series. The average correlation between the series is 0.54, with a mean segment length of 146 years due to pruning of the data and a lag-1 autocorrelation of 0.3.

The removal of non-climate related biases in tree-growth can help to increase the robustness of a chronology. This improvement can be accomplished by selecting historical series, which have been sorted to high-elevation microclimate growth characteristics by the provenance model and are therefore more likely to have a stronger temperature signal. Considering this information of the series allows for exploration of differences in MXD mean level offsets between the sites (Fig. S1). If not addressed, these differences can lead to deflated or inflated values when RCS detrending is applied (Zhang et al. 2015; Römer et al. 2021; Hartl et al. 2022), especially if a period is mainly covered with series from one elevational growth characteristics. RCS mainly requires a great sample depth (here: 352 series), a homogeneous distribution of tree-ring series in time (Fig. 2), heterogenous tree age and homogeneity in the provenance of historical and living series (Briffa et al. 1992; Düthorn et al. 2015; Esper et al. 2003; Melvin et al. 2013), which is improved by the provenance approach of Kuhl et al. (2023). The RCS detrended chronology shows more variability and a stronger negative trend towards the early nineteenth century compared to 300-year spline and Hugershoff detrending (Fig. S2a), indicating a stronger preservation of the low-frequency (Briffa et al. 1992). The standard deviation of the 100-year smoothed chronology is almost twice the size compared to other detrending methods when we apply SF age-dependent spline detrending (Fig. S2a). The SF RCS chronology, however, does not show significant differences to the classical RCS approach (Fig. S2b).

Spectrum analysis emphasizes that a smaller window of 31 years can stabilize the variance better than a wider window of 201 years. However, stabilization on narrow window can remove low-frequency signals which are related to temperature (Fig. S4a). While the 31-year window stabilization has a reduced power spectrum moving towards lower frequencies, greater windows (e.g. 201 years) preserve more low-frequency information but in turn do not stabilize the variance as well (Fig. S4b). Our new approach detangles low- from high-frequency bands by stabilizing the variance individually on both, the high- and the low-frequency sub-chronologies. Treating low- and high-frequency on different window lengths reduces the loss of low-frequency signals when applying a short window but at the same time improves stabilization compared to using a wider window for the full chronology.

### Climate-growth relationship

**Fig. 3:**
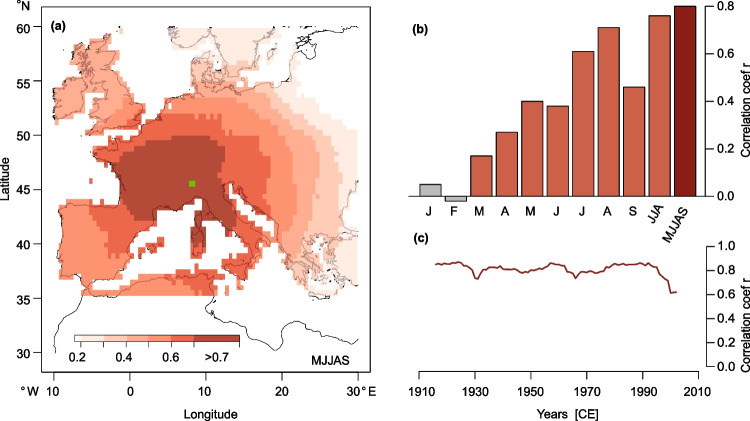
**a** Correlations between the final (combined and variance stabilized) MXD chronology and gridded mean May–September (MJJAS) temperature data (CRU TS 4.06 0.5°) from 1901–2017 CE **b** Temperature data of all grids with spatial MJJAS correlations ≥ 0.7 (**a**) are averaged to calculate monthly correlations between January and September, as well as using two different summer means (June–August JJA and May–September MJJAS). Grey colouring denotes non-significant correlations (*p* < 0.05), orange shows significant correlations and red illustrates the MJJAS window that is chosen for further analysis **c** Moving 31-year correlations between MJJAS mean temperature and the MXD chronology

Correlations between temperature anomalies and the MXD chronology reveal a positive relationship between tree-growth and temperature. Spatial correlations for MJJAS and the chronology between 1901–2017 CE show the significantly high (p < 0.05) response between Western European temperature variability and MXD indices (Fig. 3a). Grid points over France, Switzerland, Southern Germany, Northern Italy, and Western Austria reached highest correlations of r ≥ 0.7. Altogether, correlations do not drop below 0.5 in Western Europe. The ability of the Alpine chronology to represent past temperature variability in Western Europe is strengthened by calculating static correlations from 1901–2017 CE between temperatures of the grids exceeding 0.7 for MJJAS in Fig. 3a and the MXD indices (Fig. 3b). From March onwards, significant correlations (p < 0.05) with temperature are observed with highest monthly correlations for August (r = 0.71). The coherence between the proxy and instrumental temperatures increases when considering seasonal means up to r = 0.8 (MJJAS). Moving window correlations indicate a temporally stable relationship over the full instrumental period from 1901–2017 CE (Fig. 3c). Although correlations slightly decline in recent years, r values do not fall below 0.6.

### Reconstruction of Alpine summer temperature anomalies

**Fig. 4:**
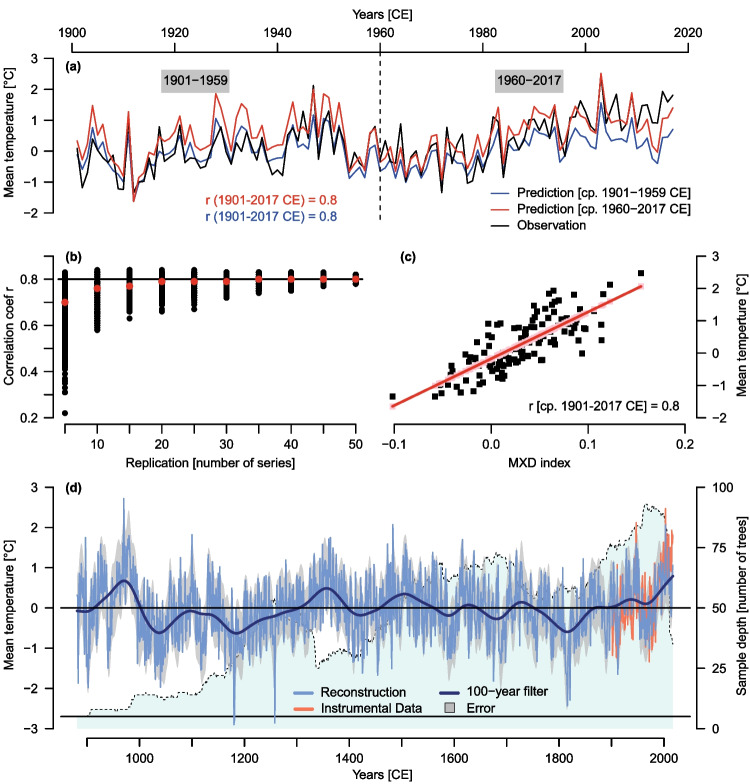
**a** Linear Regression Models between the MXD chronology and May–September mean temperature anomalies [w.r.t. 1961–1990 CE] (MJJAS Temp) of calibration periods (cp.) 1901–1959 CE (blue) and 1960–2017 CE (red). Detailed performance measures of the cross-validation are listed in Table S2 **b** The effect of replication on regression model accuracy: Correlations r between MJJAS Temp and instrumental data between 1914–2002 CE depending on different sample replications (5–50). Black dots represent correlation coefficient between the individual model runs (1000 per replication step) and instrumental data. Red dots represent the medians of the 1000 runs (r_median_). The black line shows r for the regression performed on the full period 1901–2017 (min. replication 71 samples) **c** Model fit of the linear regression between the MXD indexes and instrumental data on the full period 1901–2017 CE, which is used for the reconstruction **d** Final MJJAS Temp reconstruction: the blue line shows the reconstruction with a 100-yr smoothing filter (dark blue). Grey shading presents the error range of the reconstruction and orange the instrumental data. Light blue denotes to the sample depth n

For reconstruction, a linear regression model was chosen as these models perform best on both cross-validation periods (1901–1959 CE, 1960–2017 CE) with correlations of 0.8 on the entire period 1901–2017 CE between observed and predicted time series (Fig. 4a). These cross-validation models explain up to 70% of the variance and are stable in time with the validation periods reaching r > 0.82 (Table S2). Although both models exhibit a high level of performance, they predict underexaggerated values for the latest decades. While the model trained on the early period fails to capture the recent warming from 1995 CE onwards, the model for the later period cannot accurately predict the increase in temperatures since 2010 CE.

Uncertainties of the reconstruction are highly related to changes in replication. With decreased replication further back in time, model uncertainties are likely increasing. To test the reliability of the linear regression model depending on replication, randomly built sub-chronologies of different replications were fitted in linear regression models over a period from 1914–2002 CE, where the replication is constantly over 50 series. This smaller window was chosen to test the replication effect from 5 up to 50 series per sub-chronology. Results show a high correlation of the sub-chronologies to the instrumental data until the replication drops below 15 series (r_median_ ≥ 0.8) (Fig. 4b). When the sample replication equals ten series or lower, the linear models lose accuracy as the confidence intervals increase towards lower correlation values. Still, r_median_ does not fall below 0.7.

The robust temperature signals and the stability of the linear model with respect to replication fluctuations enable the construction of a millennium-length MJJAS temperature reconstruction. The reconstruction model fitted to 1901–2017 CE explains 64% of the variance with r = 0.8 and is used to predict and revise Alpine temperature anomalies until 881 CE (Fig. 4c, d).

The temperature reconstruction (Fig. 4d) from the European Alps displays that long-term temperature trends decrease during the early and high medieval times from 881–1200 CE. Temperatures rise again until late medieval ages in the co-called medieval climate anomaly (MCA) and peak in the 1360 s, which are on average 0.76 °C (uncertainty range: -0.08 to 1.6 °C) warmer than the reference period 1961–1990 CE. This maximum is followed by a slow long-term decrease in temperatures during the Little Ice Age (LIA) until the early nineteenth century, when the Tambora eruption caused the “year without summer” of 1816 CE (Stothers 1984; Oppenheimer 2003). This decade is the coldest decade of the record and marks the peak of the LIA. Here, temperature anomalies drop to -1.28 °C (uncertainty range -2.12 to -0.44 °C). Between the end of the MCA and the peak of the LIA, temperatures decrease in total by 2.04 °C. Afterwards temperatures rise again from 1900 until 2017 CE. This increase is shortly disrupted between 1950 and 1980 CE.

The most prominent long-term periods of cooling are between 1000–1300 and 1750–1870 CE, while warmer phases are found between 910–1000, 1300–1400, 1475–1575 and 1900–2017 CE. The 971–980 CE period represents the warmest decade of the record with a decadal average of + 1.17 °C (uncertainty range: 0.25 to 2.09 °C), followed by the 960 s, 1500 s, 980 s and 2000s. Besides the 1810s, coldest decades of the record are the 1180 s, 1040 s, 1030 s and 1170 s. The absolute warmest and coldest years of the record are not necessarily located within these decades. Warmest years are recorded in 970 (2.72 °C), 1483 (2.07 °C), 2003 (2.06 °C), 1100 (1.82 °C) and 980 CE (1.80 °C), while the coldest years are 1180 CE (-2.9 °C), 1258 (-2.86 °C), 1181 (-2.8 °C), 1816 (-2.43 °C) and 1821 CE (-2.29 °C).

## Discussion

### The new approach: eliminating interference signals from elevation and insects

The here presented new approaches aim to make reconstructions, which depend on historical series, more robust. Elevation and the potential weak temperature signals in historical series have been neglected in the past when building chronologies. Using a ML based provenance model helps to overcome this problem (Kuhl et al. 2023). The provenance of the historical series allows the selection of series, that are likely to be most sensitive to temperature, and therefore improves the temporal stability of the climate sensitivity in periods beyond the lifespans of the living trees.

As MXD increases with decreasing elevations (Zhang et al. 2015; Hartl et al. 2022), mean adjusting the series can reduce elevational biases while detrending with RCS. Lower elevation series have higher MXD levels on cambial age scale compared to higher elevations and can therefore potentially alter the regional curve towards higher mean values. This can be true for elevational differences of 100 m (Fig. S1). Microsite differences like this have already been observed and critically viewed in multiple studies in terms of regional curves biases in RCS detrending (Gunnarson et al. 2011; Düthorn et al. 2013; Hartl et al. 2021, 2022). In previous reconstructions, these potential offsets between historical series have been neglected, which is mainly due to the lack of information on the elevational origin of historical tree-ring series. Simultaneously, differing MXD values of the series can also result from temperature variability and changes in treeline (Büntgen et al. 2022a) and there is a likelihood that mean adjustment causes a loss of temperature information. However, site control of historical series offers the possibility to improve RCS detrending of living and historical series to reduce visible biases in the data due to elevational non-temperature related MXD offsets as presented in Fig. S1.

The correction of negative outliers is indispensable when chronologies are based on tree species with recurring insect outbreaks like larch (Büntgen et al. 2006; Aryal et al. 2023). As the outliers in a chronology can potentially result from volcanic cooling, the inclusion of a non-host chronology in combination with SAOD-analysis present necessary pre-steps before applying correction coefficients. This can ensure that high-frequency temperature signals are not removed from the data during this process.

The chronology depicts a different course of the temperature history when provenance information of historical series is neglected in the reconstruction process (Fig. S5). This reconstruction profits from more available samples to extend the record further until 729 (*n* = 5). Compared to the main reconstruction, this record shows higher temperature estimates in the medieval period until 1000 CE and between the twelfth-fifteenth century. A visible decrease in temperatures during the LIA is almost 150 years later than in the main reconstruction but shows a stronger decline due to the increased values from the twelfth-fifteenth century. Higher temperature estimates in this period most likely result from the inclusion of historical series from lower elevation growth characteristics with higher MXD levels and from the neglection of potential level offsets (e.g., see Hartl et al. 2022 for SV).

### Mitigated low frequency signals

**Fig. 5:**
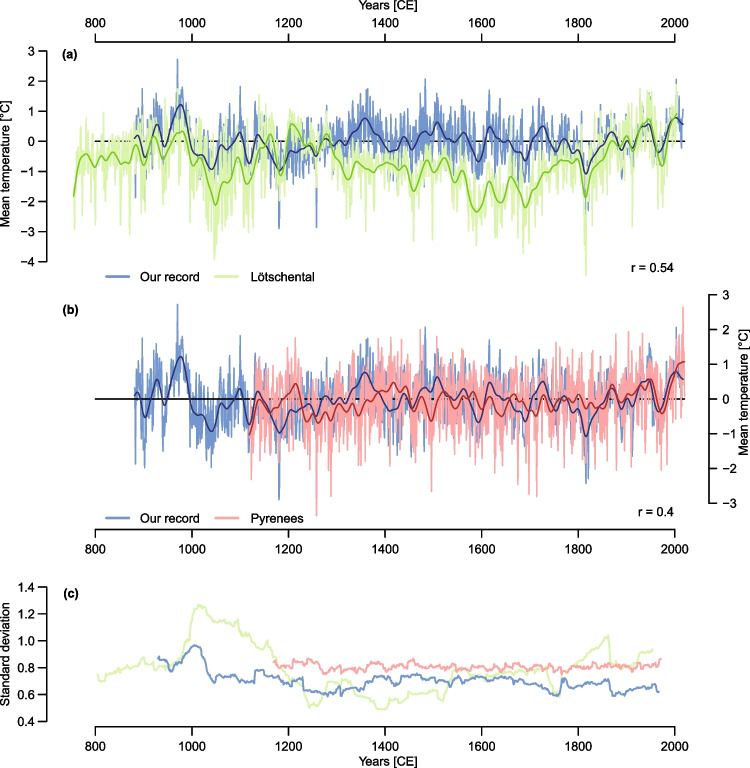
**a** Comparison between the Lötschental (green) and our record (blue) and **b** comparison between the Pyrenees (coral) and our record (blue). Dark lines in **a** and **b** present the 30-year smoothed data. r equals the correlation between both records in their common period, respectively **c** Differences in standard deviation of the Pyrenees (coral), the Lötschental (green) and the new Alpine (blue) records (100-year running window)

For almost two decades, the Lötschental reconstruction has been the state of the art record for European summer temperatures and was used in several larger network studies to present Western European temperature variability (Esper et al. 2007c; Brázdil et al. 2010; Luterbacher et al. 2016; Wilson et al. 2016; Anchukaitis et al. 2017; Torbenson et al. 2023; Wang et al. 2023). The comparison between the new reconstruction and the Lötschental record highlights some major differences between 1000–1900 CE (Fig. 5a). In contrast to the Lötschental record, the new reconstruction shows less long-term variability. Although a long-term temperature decrease during the LIA is observed, it is not as pronounced as in the Lötschental. There, the onset of the cold period begins after a peak at 1200 CE whereas the cooling in the new reconstruction starts approximately 160 years later in the mid-fourteenth century. In the Lötschental record, the drop in temperatures (w.r.t. 1901–2000 CE) between the warmest pre-LIA decade in the 1200 s (0.66 °C) and the 1810s (-2.31 °C) is 2.97 °C and the increase in temperatures between the coldest decade (1810s) and the warmest following decade of the 1940s is 3.08 °C. The reconstructed temperature decrease since the end of the MCA is almost a degree less in the new record and could hint that the European Little Ice Age temperature decrease was not as strong as the Lötschental record estimated and that the reconstruction implies that summer temperatures are not as cool as in the period between 1000 and 1250 CE. However, there is evidence that the LIA must have existed in Europe, for example by historical records or glacier advances in the Swiss Alps (Grove 2001), which challenges the reconstruction’s skill in low-frequency variation.

We tested and eliminated various potential sources of error during the development of the reconstruction (see Table S3 for more detailed information). Sources, which can alter the low-frequency signals, include the distribution of tree age in time, the detrending method, the selection of series or an inadequate classification by the ML models (Kuhl et al. 2023). For example, the model for MV uses data of only two different elevations as reference and despite the model performance score (f1-score) of 0.99, series might align more with patterns from lower elevations but will be sorted to either of the two elevations. We tackle this problem by selecting only those series that are sorted to an elevational class with a prediction probability greater than 0.8. As mentioned before, the mean adjustment might alter climate signals, when tree-ring series are adjusted to higher elevation MXD levels. However, excluding these steps (provenancing and mean adjustment) does not increase the low-frequency signal, especially not during the LIA (Fig. S5).

Besides the difference in the low-frequency signal in both records, the variance of the new record is more stable in time (Fig. 5c). In the Lötschental record, however, the variance is not as stable and shows stronger temporal fluctuations, especially before the thirteenth century CE. As variance changes are likely artificial and an artefact of alternating replication or varying ecological habitats, the increase in variance pre 1200 CE might result from a change in replication and the different origin of this historical tail being exclusively composed of the Simplon valley (Büntgen et al. 2006; Esper et al. 2016). Despite these differences, the correlation in the common period (881–2004 CE) between both records is 0.54 and 0.62, when a 500-year high-pass filter is applied to the Lötschental record. It indicates that albeit low-frequency signals are mitigated in the new reconstruction higher frequency signals are more coherent.

A recently developed reconstruction from the Pyrenees (Büntgen et al. 2024), however, coincides with the new Alpine record (Fig. 5b). Both reconstructions show a similar low-frequency variability. They correlate with 0.4 but correlations reach 0.74 when both chronologies are 300-year low-pass filtered. Although the LIA period is weakly pronounced in both reconstructions, the Pyrenees record does not record a strong cooling in the 1810s, which is prominent in most Alpine records (Table 1) (Schweingruber et al. 1987, 1988; Büntgen et al. 2005, 2006; Frank and Esper 2005b; Corona et al. 2010, 2011; Trachsel et al. 2012; Coppola et al. 2013; Leonelli et al. 2016). The standard deviation of the Pyrenees record in Fig. 5c is stable with lower variation than in the Lötschental record or the record of this study.

A focus on the warm and cold decades of the Lötschental and the new record reveals that while both reconstructions agree on the 1810s as the coldest decade, the warmest decade in the new record is not in the last decades but earlier in the 970 s. The most recent decades, which are the absolute warmest decades according to the Lötschental record, are not the warmest in the new record anymore. Here, the warmest decades are mainly between 960 – 990 CE. Nonetheless, both records show the 2000s as one of the five warmest decades. Surprisingly, the new record does not estimate the 2010s to be among the warmest decades, which is not in line with the instrumental data.

### Revising cold and warm extremes in Alpine temperature history

**Fig. 6:**
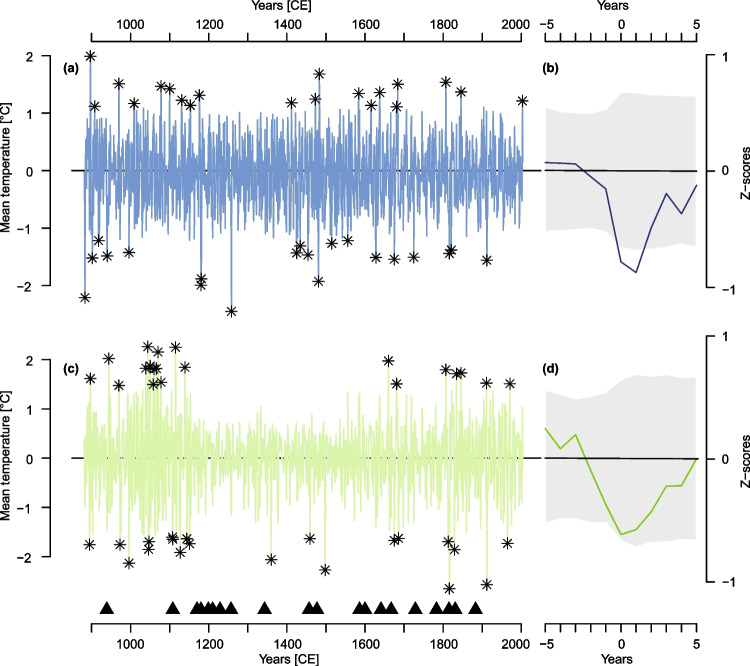
Extreme Year Analysis: 30-year high-pass filtered reconstructions from this study in **a** and Lötschental in **c**. Asterisks denote to the 20 warmest and the 20 coldest years, respectively. Triangles show 20 strongest volcanic events between 880–2000 AD (see Table S4). For these 20 events, panels **b** and **d** show the Superposed Epoch Analysis (lag = 5, residuals from the 5 years prior to event) with mean (black line) and 99% confidence intervals (grey) after bootstrap resampling (n = 10,000)

A stable and unbiased variance is crucial for robust extreme year analysis (Frank et al. 2007). Artificial fluctuations of variance can lead to falsely detected extreme events and consequently alter interpretations of extreme year distributions. This influence becomes visible when 30-year high-pass filter reconstructions and their top 20 extreme years are compared (Fig. 6a and c, Table 2)**.** The asterisks in the plots denote the 20 warmest and coldest years of the records, respectively. If variance is not stable in time (Fig. 5b), extreme years like volcanic cooling events are accumulated in periods with increased variance, for example the 11-twelfth century in the Lötschental record. As these volcanic induced cooling years are recorded by tree-ring proxies like MXD, they are frequently used to detect volcanic events (Esper et al. 2017) and reveal information on their impact on temperatures and on their link to societal developments like mass migrations, famines or cultural heydays (Büntgen et al. 2020). A bias in variance can thus alter volcanic event detection and interpretation. Although the Lötschental record detects some of the strongest 20 volcanic SAOD events during the last millennium, a SEA of these events reveals that a highly significant (p < 0.01) cooling in the year of the event and year after the event is not detectable in this record (Fig. 6d). In comparison, the new reconstruction has a temporally more stable variance (Fig. 5b), and the event year as well as following years are found to be significantly cooler than the five years prior to the event (Fig. 6b). As most volcanic events of the SEA fall within periods of lower variance in the Lötschental record, the averaged cooling of these events does not exceed the non-volcanic induced declines in periods when the variance is higher. Further, the growth decline relative to the years prior to the event is less pronounced than in the new record. With fluctuating variance, the volcanic induced cooling years in the Lötschental record are either no outliers in periods of low variance or surrounded by outliers in periods of higher variance. The residuals between the event year temperatures and the reference period are consequently lower.

**Table 2:**
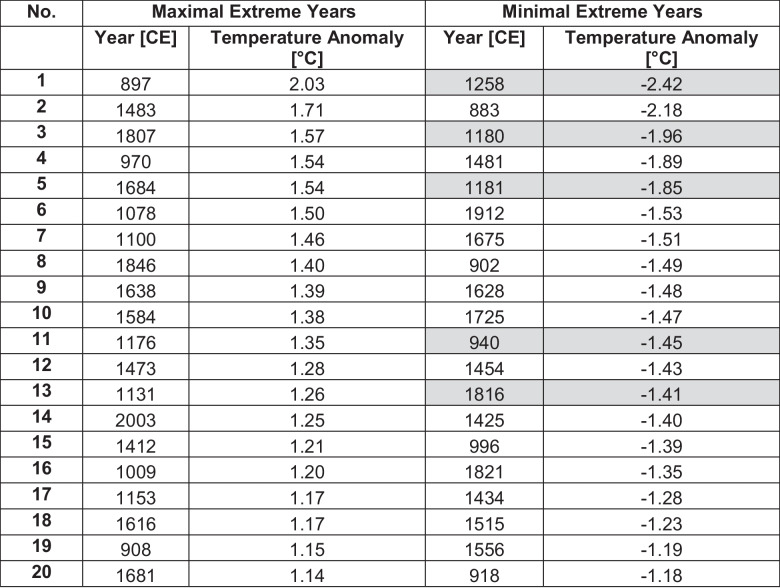
Top 20 minimum and maximum extreme years (descending order) of the new Alpine record between 881–2004 CE. Highlighted entries align with an eruption year or the year after using the dates of Table S4

An example of the influence of variance on the interpretation of extreme events is given by focusing on the Samalas eruption in 1257 CE, which is the largest volcanic eruption of the last millennium (Lavigne et al. 2013). The following volcanic cooling is recorded in both reconstructions with -2.32 °C in the new record and -1.18 °C in the Lötschental record (w.r.t. 1252–1256 CE), respectively. While the Samalas induced cooling in 1258 CE presents the strongest negative extreme event in the new record, it does not fall under the extreme events detected in the Lötschental record. This might be explained by variance fluctuations in the high-frequency domain. The resulting loss of climate information might explain why this eruption is not imprinted in the top 20 cold events of the record. Guillet et al. (2017) present historical evidence for bad (grape) harvest, increased prices, heavy precipitation and floodings in the regions of Western European region, where temperatures correlate high with the new record (Fig. 3a). A strong cooling in 1258 CE can be observed in the Pyrenees record being -3.26 °C colder than the average of the previous five years (see Fig. 5b, Büntgen et al. 2024). Other NH and Western Europe reconstructions record decreases between -0.55 to -1.8 °C compared to the ten year mean prior the events (here: -2.52 °C) (Esper et al. 2013; Schneider et al. 2015; Stoffel et al. 2015; Wilson et al. 2016; Hartl-Meier et al. 2017a). Less pronounced cooling in these records compared to the Pyrenees or the Alps is likely due to the observed spatially diverse intensity of the post volcanic cooling (Guillet et al. 2017; Büntgen et al. 2022b). For the new Pyrenees and Alpine reconstructions, the 1258 CE cooling is unrivalled in the last millennium (Figs. 5b, S7). In both, the cooling following the Samalas eruption is stronger than the cooling observed for 1816 CE after Tambora, which is less prominent in the Pyrenees record compared to the Alpine region. The new record agrees with previous studies that showed that summer temperatures in the 1800/1810s have been relatively cold already before the event compared to the situation in the 1250 s (e.g., Frank and Esper 2005b; Corona et al. 2010; Trachsel et al. 2012; Schneider et al. 2015). Although the volcanic induced decrease of 1.01 °C compared to the five years prior to the event appears less severe than the Samalas induced temperature decline, the impact of the 1810s CE cooling on humanity was strong which is proven by historical evidence (e.g., Brázdil et al. 2017; Büntgen et al. 2020). We are aware that the LBM correction can bias the strength of volcanic cooling which is mediated by the reconstruction. In years where LBM events and volcanic events align, LBM induced density decline can amplify the reconstructed compared to the true volcanic cooling. However, as the extreme year 1258 CE is also detected in the new Pyrenees record by Büntgen et al. (2024), which is based on LBM-unaffected pine trees (*Pinus uncinata* Ramond s. str.), it supports our findings that people in Western Europe experienced a year of unusual cold summer temperatures in 1258 CE.

The top 20 summer heat extremes in the new record are relatively balanced throughout the covered period with clusters in the late medieval times and during LIA. Although long-term temperatures during the LIA are decreasing, exceptionally warm summers relative to surrounding decades are observed in a recent study (Wanner et al. 2022). For example, the summer in 1473 CE was extremely hot and dry in Europe causing early (grape) harvests or harvest failures, loss of transportation routes (rivers), water shortages and wildfires (Camenisch et al. 2020). Likely due to a decrease in variance in this period, this year is not observed as an extreme year in the Lötschental record. Similarly, the 2003 CE heatwave appears as an extreme year when long-term trends are included (Büntgen et al. 2006), but not when the record is high-pass filtered. From 2003–2017 CE no cool extremes are observed in the new Alpine record but no heat extremes either which does not agree with instrumental data. Resuming the fit of the reconstruction model (Fig. 4a) this might result from the models’ limitation to fully capture the instrumental warming after 2010 CE.

### Is there a divergence problem in the Alps?

Although the fit between instrumental and proxy data (r = 0.8) is very high, the dataset shows signs of a beginning divergence from 2010 CE onwards (Fig. S7). The divergence phenomenon describes the weakening of the linear relationship between temperature records and tree-ring proxies (TRW and MXD) during the twentieth century (D’Arrigo et al. 2008). This phenomenon has been observed in multiple sites all over the Norther Hemisphere (Wilmking et al. 2005; Wilson et al. 2007; D’Arrigo et al. 2008; Andreu-Hayles et al. 2011; Shah and Shah 2015; St. George and Esper 2019) and in low elevation sites in the European Alps (Corona et al. 2010). However, divergence has not been detected in high elevation sites in this region during the twentieth century for European larch (*Larix decidua* Mill.) (Büntgen et al. 2008). Here, we find first indications of a developing divergence phenomenon in the Swiss Alps in larch. From 2010 CE onwards MXD does not follow the increased warming observed in instrumental data. This is underlined by a decline in the 30-year running correlations between temperature and MXD indices from 1990 CE until present in Fig. 3c. The linear relationship is weakened and consequently, the prediction performance of linear regression likely decreases with increasing divergence. Correlations with other climate parameters, for example drought or precipitation, do not show a shift in signal in the Alps yet, but might in the future as it has been observed in other regions, for example Corsica (Römer et al. 2021). There, drought, and consequently summer precipitation become more important for limiting tree growth. Precipitation signals are, however, difficult to assess in network datasets like the new Alpine chronology since precipitation patterns in mountainous regions can vary strongly between valleys and elevations (Sevruk 1997; Auer et al. 2001; Schmidli et al. 2002).

## Conclusion

In this study, we present a new temperature reconstruction based on tree-ring data from the Swiss Alps. We introduce a new approach to improve temperature reconstructions and their temporal reliability by considering elevational differences among data from historical buildings, removing LBM signals with improved methods, and applying novel approaches to stabilize variance. Albeit low-frequency trends are muted in our new reconstruction compared to previous records, high-frequency temperature variability shows strong agreements with large volcanic eruptions which is in line with historical evidence. We show how artificial variance increases may alter extreme year analysis including the 1257 CE Samalas eruption, which led to stronger regional cooling than the 1815 CE Tambora eruption. Our study thereby emphasizes how decision making, data selection and treatment in dendroclimatology influences the outcome of a reconstruction approach (Büntgen et al. 2021). The different applied methods, in particular the (1) data selection (of proxy and target), (2) mean adjustment, (3) LBM correction, (4) variance stabilization, and (5) the chosen method of reconstruction, alter the resulting record and interpretation of temperature variability towards its extreme events and long-term temperature signals. This study highlights the urgency to continue the development and testing of new methods to improve the temporal stability and reliability of climate reconstructions. It stresses the importance that with new methods revising existing records to improve our knowledge about past climate variability is indispensable.

## Supplementary Information

Below is the link to the electronic supplementary material.

Supplementary file1 (PDF 1278 KB)

## Data Availability

Raw tree-ring measurements will be submitted to the International Tree Ring Database (ITRDB).
