# Supplementary material for: Revising Alpine summer temperatures since 881 CE
Source: Clim Dyn. 2024 Mar 22;62(7):6141–57. doi: 10.1007/s00382-024-07195-1 (PMC11420296; doi:10.1007/s00382-024-07195-1)
Supplement: Supplementary file 1 — Supplementary file1 (PDF 1278 KB) [file 382_2024_7195_MOESM1_ESM.pdf]

Supplementary Material

Submitted to Climate Dynamics

Revising Alpine summer temperature history since 881 CE

Eileen Kuhl[1], Jan Esper[1,7], Lea Schneider[2], Valerie Trouet[3], Marcel Kunz[1], Lara Klippel[4], Ulf Büntgen[5,7-9] and Claudia Hartl[6]

[1] Department of Geography, Johannes Gutenberg University, Mainz, Germany

[2]Department of Geography, Justus-Liebig-University, Gießen, Germany

[3]Laboratory of Tree-Ring Research, University of Arizona, Tucson, USA

[4]Deutscher Wetterdienst, Offenbach, Germany

[5]Department of Geography, University of Cambridge, Cambridge, UK

[6]Nature Rings - Environmental Research and Education, Mainz, Germany

[7]Global Change Research Centre (CzechGlobe), Brno, Czech Republic

[8]Swiss Federal Research Institute (WSL), Birmensdorf, Switzerland

[9]Department of Geography, Masaryk University, Brno, Czech Republic

Corresponding author: Eileen Kuhl (eikuhl@uni-mainz.de), Johann-Joachim-Becher Weg 32, 55128 Mainz, Germany, Orchid: 0000-0002-1246-6030

Includes:

Tables S1-3

Figures S1-8

Table S1 Data Selection after applying the Provenance Model to Simplon and Zermatt/ Zmutt historical series (Kuhl et al. 2023). Numbers include living and historical series.

	Simplon Valley			Matter Valley			
**Site (Elevation [m asl])**	**SV1 (1900)**	**SV2 (2000)**	**SV3 (2200)**	**MV1 (2000)**	**MV2 (2300)**	**MV3 (2300)**	**SUM**
**Total number of series**	56	24	25	180	24	43	**352**

Table S2 Performance measures (correlation between predicted and observed (r), explained variance (R^2) and root mean squared error (RMSE)) of the linear models in **Figure 6a)**. Asterisks show the columns for the linear model calibrated on the early period, without asterisks are the columns for the linear model calibrated on the later period.

	Calibration Periods		Validation Periods	
**Period [CE]**	**1901-1959***	**1960-2017**	**1960-2017***	**1901-1959**
**r**	0.83	0.84	0.84	0.83
**R^2**	0.69	0.7	0.7	0.69
**RMSE**	0.39	0.46	0.7	0.64

Table S3 Testing for low frequency signals: Approaches applied to analyse the missing low frequency in our reconstruction.

**No**	**Approach**	**Visual improvement of low-frequency signal?**
**1**	No mean adjustment	No
**2**	Including another high elevation living site.	No
**3**	Other detrending methods (Spline, Hugershoff, signal-free age-dependent Spline signal-free RCS, see Fig. S2)	No
**4**	Include low elevation historical series and adjust their mean levels accordingly	No
**5**	Use all historical series neglecting the new altitudinal approach (see Fig. S5)	No
**6**	Subsampling (50/50 ratio Simplon/ Zermatt samples)	No
**7**	Reducing samples of houses with overrepresentation in a time period	No
**8**	Influences of tree age distribution in time	No
**9**	Only using only the historical series from Simplon	No (replication too low)

Table S4 20 volcanic events between 880-2000 CE linked with the highest volcanic stratospheric aerosol optical depth (SAOD) values in the Westwind Zone (30 - 60°N) from Sigl et al. (2021)[1]. Eruption year and volcanos were taken from Wang et al. (2022)[2] and Büntgen et al. (2020)[3]. Greyed event no. 20 is taken from Wang et al. (2022) to extend the record into the 19th century (SAOD Peak was calculated over 30-90°N[4]).

No.	Eruption Year [CE]	SAOD Peak Year[1] [CE]	SAOD Peak 30-60N[1]	Volcano
1	1257[2,3]	1258	0.46	Samalas[2,3]
2	939[3]	939	0.43	Katla[3]
3	1180[2]	1181	0.34	UE[2] /Katla[3]
4	1783[2,3]	1783	0.32	Laki[3]
5	1457[2]	1458	0.26	UE[2,3]
6	1815[2,3]	1815	0.26	Tambora[2,3]
7	1229[2]	1229	0.24	UE[2,3]
8	1641[2,3]	1642	0.23	Parker[2,3]
9	1831[2,3]	1832	0.2	UE[3]
10	1108[3]	1109	0.16	UE[3]
11	1477[2,3]	1477	0.16	Veidivötn[2,3]
12	1199	1199	0.15	UE
13	1170[2]	1171	0.14	UE[2,3]
14	1600[2,3]	1600	0.13	Huaynaputina[2,3]
15	1210[2,3]	1209	0.12	Katla[2]
16	1343[2]	1344	0.12	UE[2]
17	1586[2]	1587	0.12	Kelud[2]
18	1667[2]	1666	0.12	Shikotsu[2]
19	1729[3]	1729	0.12	UE[3]
20	1883[2,3]	1883	0.12[4]	Krakatau[2,3]

Figures

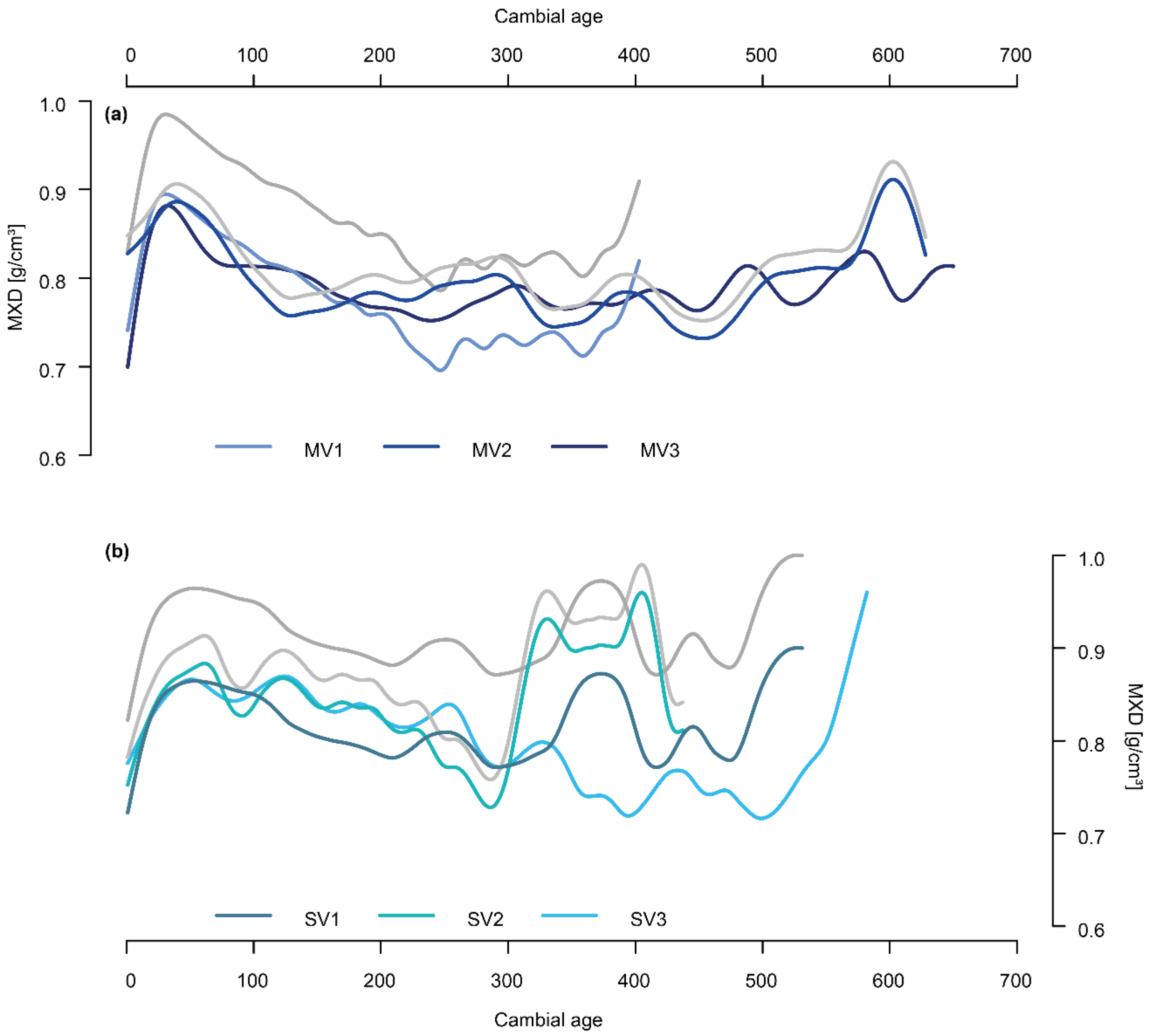

Figure S1 Mean adjustment of the Matter valley in **a** and Simplon valley in **b** depending on the regional curves. Lower elevation sites were adjusted to the highest one. Differences were calculated over the period of replication ≥ 15. Grey lines show the original curves of the sites

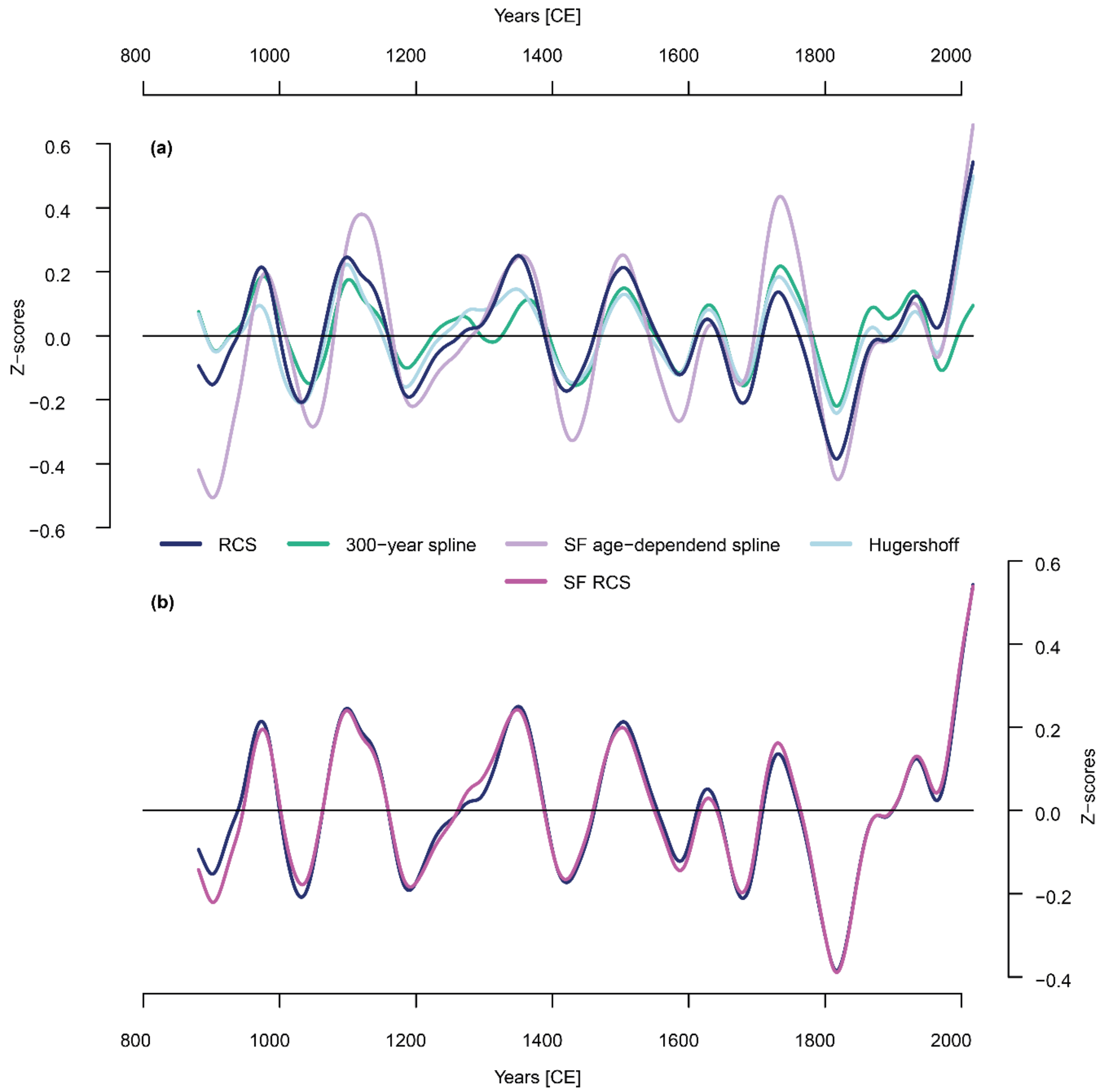

Figure S2 Different detrending methods compared with each other, smoothed with a 100-year smoothing spline. **a** Regional Curve Standardization (RCS, Briffa et al. 1992), 300-year Spline detrending (Cook and Peters 1981), Hugershoff detrending (Cook et al. 1990) and a signal-free (SF) age-dependent spline detrending (Melvin and Briffa 2008) **b** Comparison of classical RCS with the SF RCS (Melvin and Briffa 2014) detrending.

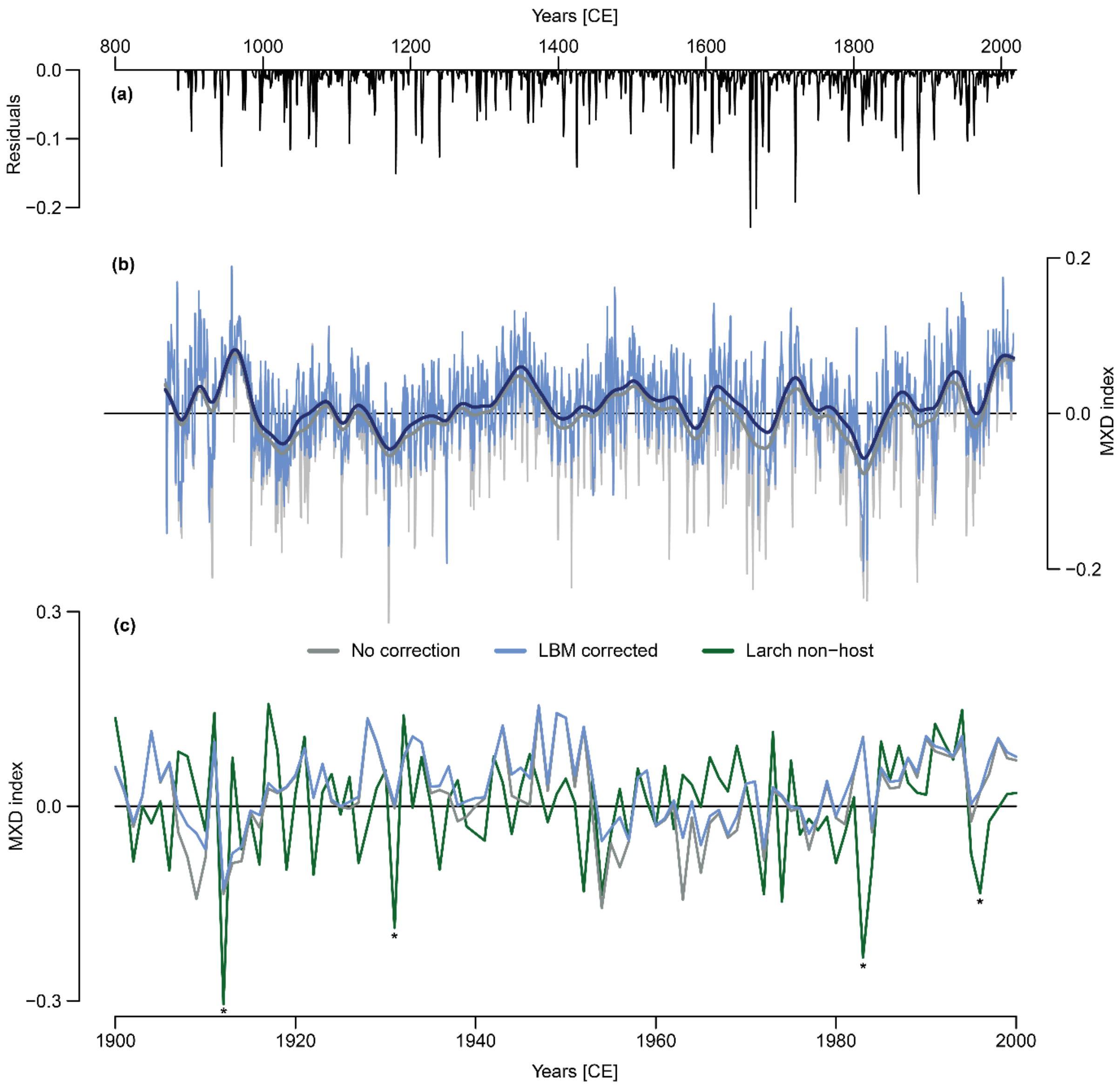

Figure S3 Larch budmoth (LBM) detection and correction using Impulse Indicator Saturation (IIS) after Pretis et al. (2016). Residuals between the original and the corrected chronology in **a** present a frequent detection of LBM events throughout the timeseries **b** The resulting corrected chronology **c** A zoom into the 20th century in strengthens how a larch non-host as addition indicator is used by the algorithm to exclude potential climate related declines (asterisks) from the correction

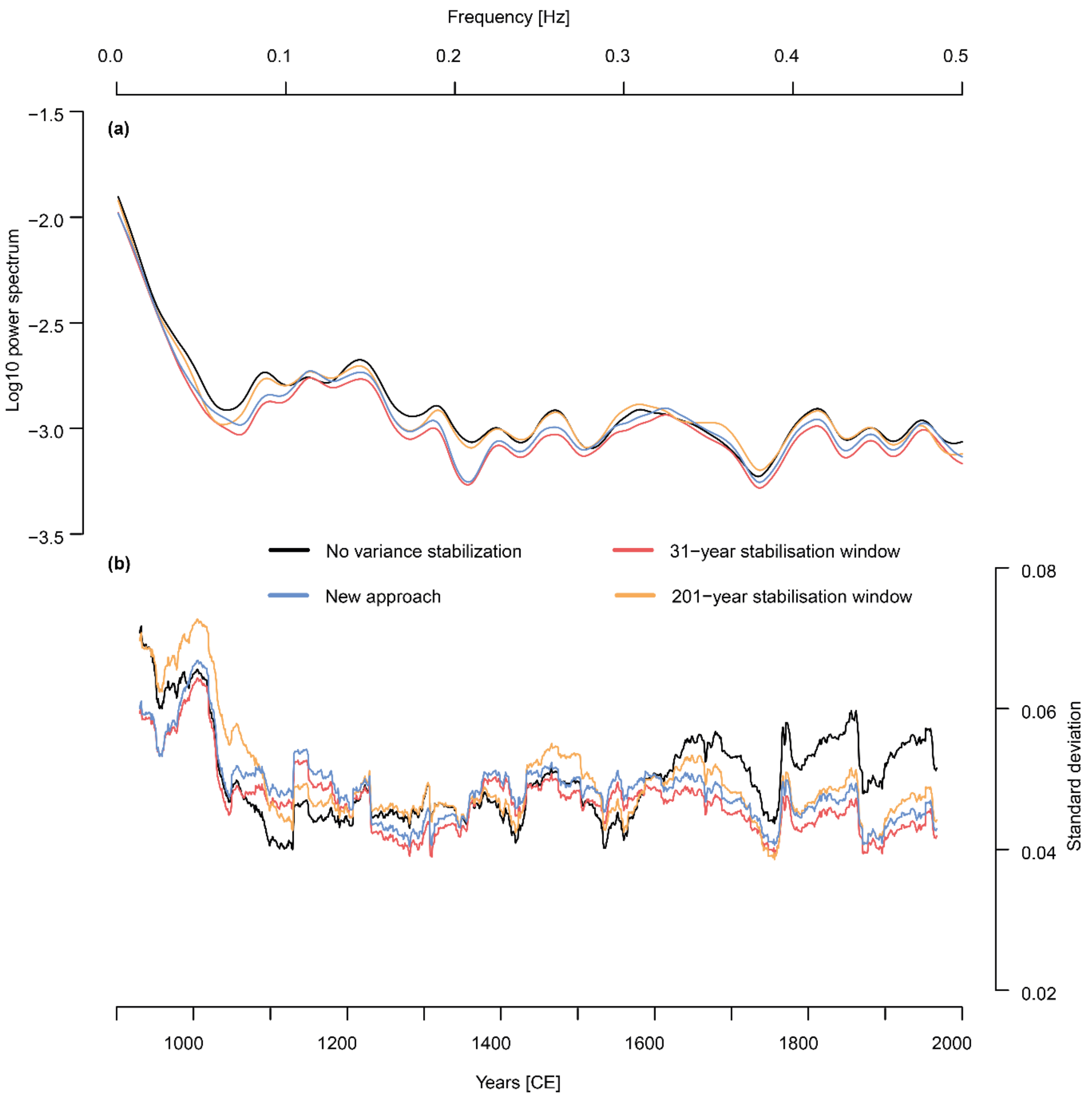

Figure S4 Variance stabilization effects of window-size and the new presented split-window stabilization using the method of Osborn et al. (1997): Panel **a** shows the spectrum analysis (log10 power spectrum) calculated using Fast Fourier Transformation. Resulting standard deviations of the chronologies are found in panel **b** and were calculated over a 100-year running window with a 1-year lag

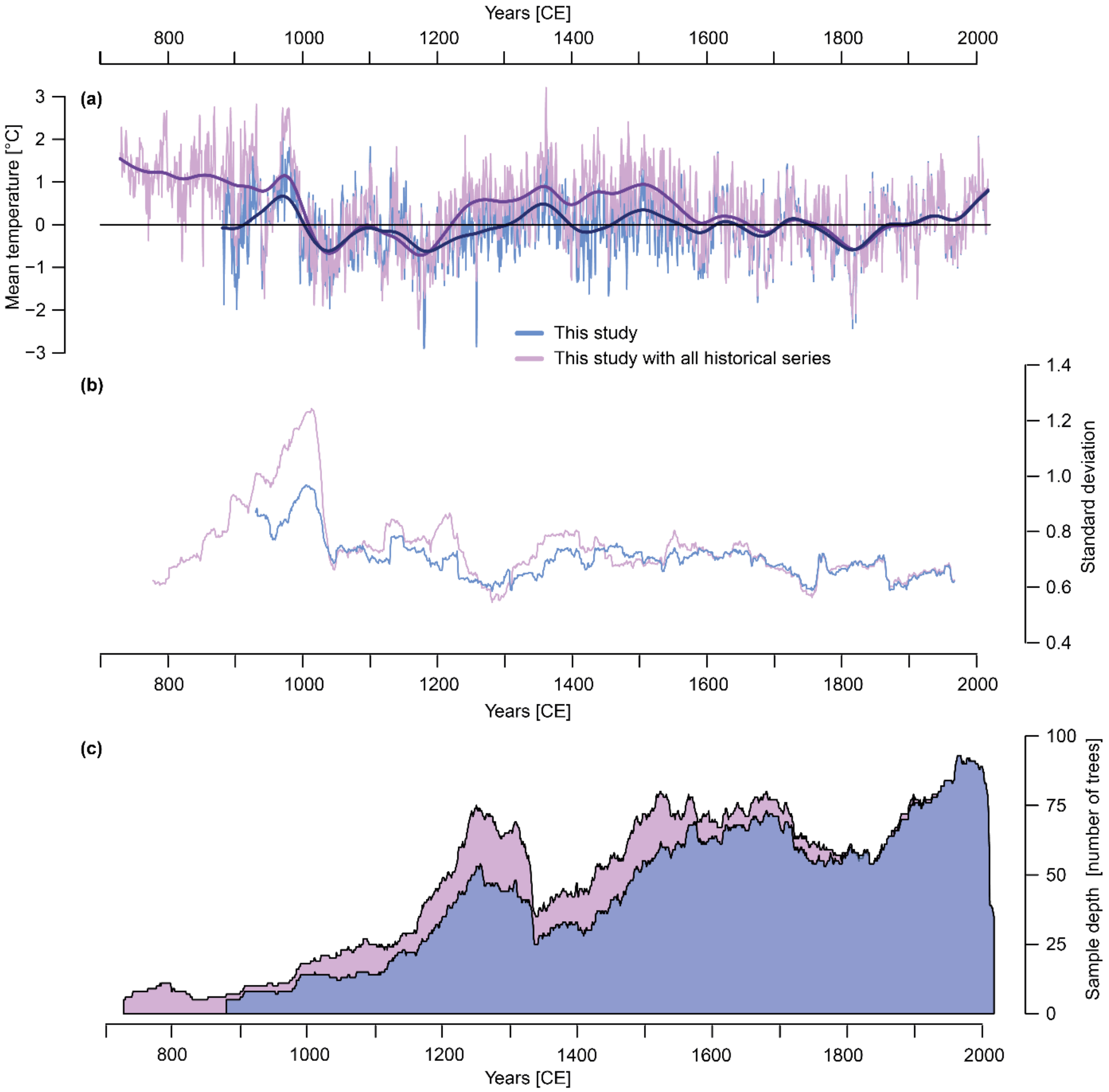

Figure S5 Comparison between a classical reconstruction (see methods chapter for more details on procedure) and the new altitude considering approach in **a**. Panel **b** shows the standard deviations of the reconstructions over a 100-year running window (lag 1) **c** presents the difference in sample depth between an altitude adjusted and a classical dataset

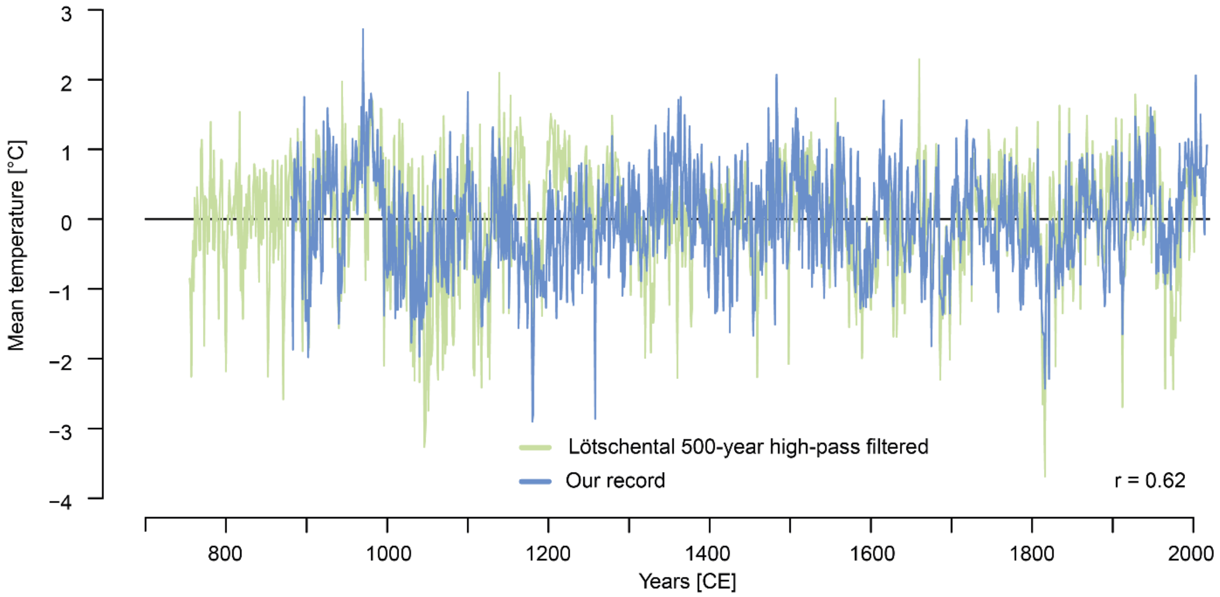

Figure S6 Comparison between here presented reconstruction and the 500-year high-pass filtered Lötschental reconstruction (Büntgen et al. 2006)

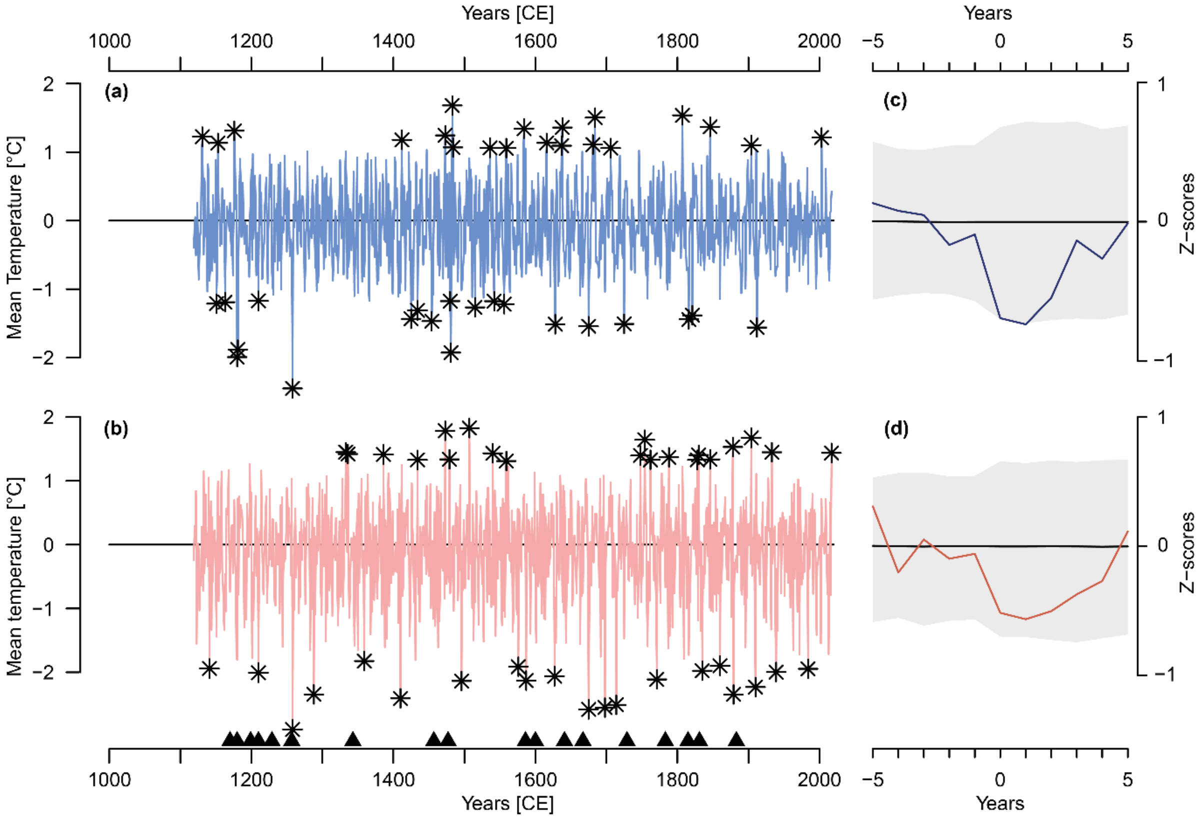

Figure S7 a 30-year high-pass filtered reconstruction from this study and **b** 30-year high-pass filtered Pyrenees record. Asterisks denote to the 20 warmest and the 20 coldest years, respectively. Triangles show 18 strongest volcanic events between 1119-2017 AD (see table S4). **c and d** Superposed epoch analysis for these 18 events (lag = 5, residuals from the 5 years prior to event) with mean (black line) and 99% confidence intervals (grey) after bootstrap resampling (n = 10,000)

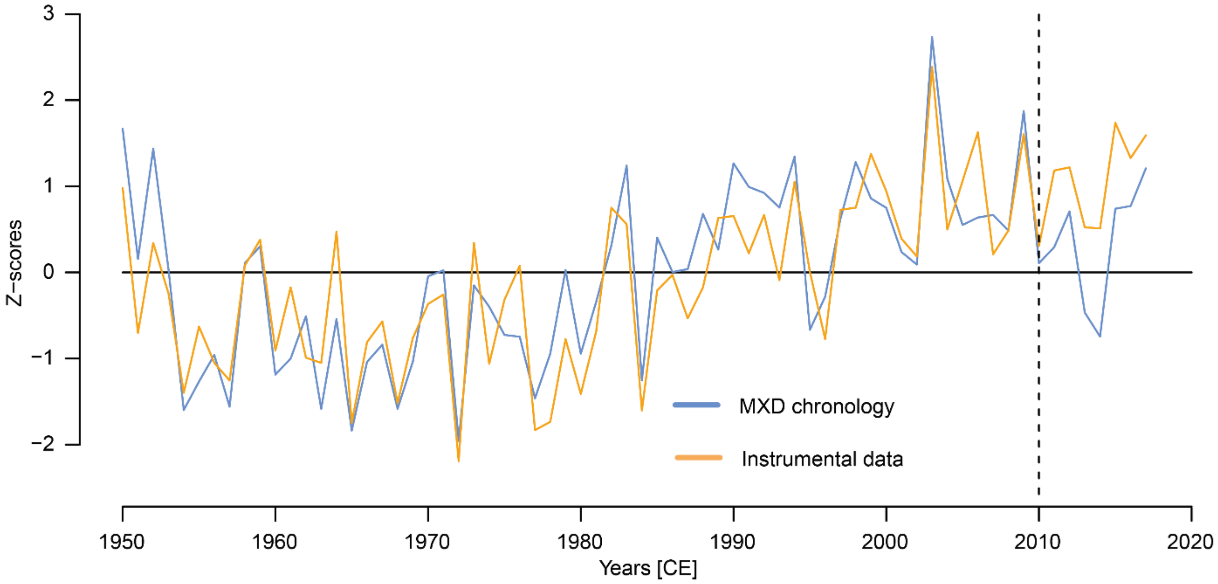

Figure S8 Z-scores of instrumental data versus the detrended MXD chronology

References

Briffa KR, Jones PD, Bartholin TS, Eckstein D, Schweingruber F, Karlén W, Zetterberg P, Eronen M (1992) Fennoscandian summers from ad 500: temperature changes on short and long timescales. Clim Dyn 7(3):111–119. https://doi.org/10.1007/BF00211153

Büntgen U, Arseneault D, Boucher E, Churakova (Sidorova) OV, Gennaretti F, Crivellaro A, Hughes MK, Kirdyanov AV, Klippel L, Krusic PJ, Linderholm HW, Ljungqvist FC, Ludescher J, McCormick M, Myglan VS, Nicolussi K, Piermattei A, Oppenheimer C, Reinig F, Sigl M, Vaganov EA, Esper J (2020) Prominent role of volcanism in Common Era climate variability and human history. Dendrochronologia 64:125757. https://doi.org/10.1016/j.dendro.2020.125757

Büntgen U, Frank D, Nievergelt D, Esper J (2006) Summer Temperature Variations in the European Alps, a.d. 755–2004. J Clim 19(21):5606–5623. https://doi.org/10.1175/JCLI3917.1

Cook ER, Briffa KR, Shiyatov SG, Mazepa V (1990) Tree-ring standardization and growth-trend estimation. In: Cook ER, Kairiukstis LA (eds) Methods of Dendrochronology: Applications in the Environmental Sciences. Kluwer Academic Publishers, Dordrecht, pp 104–123

Cook ER, Peters K (1981) The smoothing spline: a new approach to standardizing forest interior tree-ring width series for dendroclimatic studies. Tree-Ring Bull 41:45–53

Kuhl E, Zang C, Esper J, Riechelmann DFC, Büntgen U, Briesch M, Reinig F, Römer P, Konter O, Schmidhalter M, Hartl C (2023) Using machine learning on tree-ring data to determine the geographical provenance of historical construction timbers. Ecosphere :1–14. https://doi.org/DOI: 10.1002/ecs2.4453

Melvin TM, Briffa KR (2008) A “signal-free” approach to dendroclimatic standardisation. Dendrochronologia 26(2):71–86. https://doi.org/10.1016/j.dendro.2007.12.001

Melvin TM, Briffa KR (2014) CRUST: Software for the implementation of Regional Chronology Standardisation: Part 1. Signal-Free RCS. Dendrochronologia 32(1):7–20. https://doi.org/10.1016/j.dendro.2013.06.002

Osborn TJ, Briffa KR, Jones PD (1997) Adjusting variance for sample-size in tree-ring chronologies and other regional mean timeseries. Dendrochronologia 15:89–99

Pretis F, Reade J, Sucarrat G (2016) General-to-specific (GETS) modelling and indicator saturation with the R package gets. Department of Economics Discussion Paper Series University of Oxford

Sigl M, Toohey M, McConnell JR, Cole-Dai J, Severi M (2021) HolVol: Reconstructed volcanic stratospheric sulfur injections and aerosol optical depth for the Holocene (9500 BCE to 1900 CE). PANGAEA. https://doi.org/10.1594/PANGAEA.928646

Wang F, Arseneault D, Boucher É, Gennaretti F, Yu S, Zhang T (2022) Tropical volcanoes synchronize eastern Canada with Northern Hemisphere millennial temperature variability. Nat Commun 13(1):5042. https://doi.org/10.1038/s41467-022-32682-6
